# Invisible Security Printing on Photoresist Polymer Readable by Terahertz Spectroscopy

**DOI:** 10.3390/s17122825

**Published:** 2017-12-06

**Authors:** Hee Jun Shin, Min-Cheol Lim, Kisang Park, Sae-Hyung Kim, Sung-Wook Choi, Gyeongsik Ok

**Affiliations:** 1 Research Group of Food Safety, Korea Food Research Institute, 245, Nongsaengmyeong-ro, Iseo-myeon, Wanju-gun, Jeollabuk-do 55365, Korea; Shin.Hee-jun@kfri.re.kr (H.J.S.); mclim@kfri.re.kr (M.-C.L.); Park.Ki-sang@kfri.re.kr (K.P.); swchoi@kfri.re.kr (S.-W.C.); 2 Department of Molecular Science and Technology, Ajou University, Suwon 16499, Korea; ksh772211@gmail.com

**Keywords:** terahertz spectroscopy, anti-counterfeiting, security, photoresist material

## Abstract

We experimentally modulate the refractive index and the absorption coefficient of an SU-8 dry film in the terahertz region by UV light (362 nm) exposure with time dependency. Consequently, the refractive index of SU-8 film is increased by approximately 6% after UV light exposure. Moreover, the absorption coefficient also changes significantly. Using the reflective terahertz imaging technique, in addition, we can read security information printed by UV treatment on an SU-8 film that is transparent in the visible spectrum. From these results, we successfully demonstrate security printing and reading by using photoresist materials and the terahertz technique. This investigation would provide a new insight into anti-counterfeiting applications in fields that need security.

## 1. Introduction

In the last few decades, security, forgery, and alteration problems have become critical issues for many areas such as national, industrial, and individual security. Security threats can result in serious crimes and huge damage to a person or country. A representative example of this is currency counterfeiting. The result of currency counterfeiting causes currency devaluation, which can depress the economic process severely. Accordingly, the increasing trend in counterfeiting causes huge damage to the industrial economy of many establishments and significant deterioration of relations between nations. In the last decade, millions of dollars have been spent to expose fake currencies by many governments and industries. For these reasons, several scientists have struggled to solve the problem of currency counterfeiting, and they have achieved useful applications using techniques such as the holographic method or visible luminescent ink [1,2,3,4,5]. In addition, commercial branded goods manufacturers and pharmaceuticals have adopted anti-forgery techniques to distinguish an imitation from an original [6,7]. To prevent fake passports, many technologies have been studied [8,9,10]. Security is also an important issue for food industries where techniques such as oxygen sensing using a colorimetric indicator are employed in packaging [11]. This is called intelligent packaging.

Nowadays, security and anti-forgery techniques have become more advanced and simpler. These techniques—such as security ink, simple marker, plasmonic security labels, and holographic techniques [5,12]—are practically applicable, and these technologies provide the solution for security and protection against forgery problems. However, these techniques still have some problems. Using chemical materials for ink and marker can cause chemical toxicity. It is possible to mimic or duplicate other techniques. Moreover, complicated techniques would be economically infeasible.

Currently, terahertz technology has emerged as one of the techniques for security and anti-forgery. Ever since terahertz time-domain spectroscopy was developed using a femtosecond laser, terahertz technology has been attractive because it has several advantages, such as non-invasiveness, non-destructiveness, and non-ionization measurement. Owing to these advantages, terahertz techniques are considered a good approach toward security and anti-forgery. Consequently, during the last few decades, terahertz techniques have been successfully applied in security and anti-forgery applications for military applications [13,14], security screening applications [15], hidden object detection [16], artworks [17], pharmaceuticals [14], etc. Additionally, terahertz waves can be used for data security communications [18] and imaging [19].

In this study, we introduce security printing in the form of invisible information coding on photoresist materials by using terahertz time-domain spectroscopy and terahertz imaging technique. Our investigation revealed that the terahertz characteristics of SU-8 as one of the photoresist polymers are significantly changed after ultraviolet (UV) light illumination due to chemical change. SU-8 is chemically stable against many acids and bases, and is highly transparent under near-UV and visible light [20]. However, when SU-8 is exposed to UV light, this material would be sensitive to terahertz waves. Hence, the encoded information is readable with terahertz waves but transparent in the visible spectrum. Thus, the security printing code has been successfully realized in the terahertz region. This study can provide new applications for all areas that need security or innovative anti-forgery with a simple approach by using THz time-domain spectroscopy.

## 2. Materials and Experiment

### 2.1. THz Security Printing Material: SU-8

**Figure 1:**
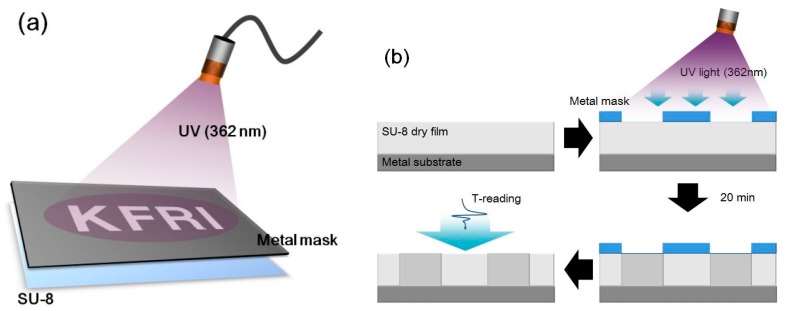
(**a**) Scheme of the UV exposure on the SU-8 photoresist and (**b**) cross-linking processing after UV illumination.

We used commercially available SU-8 dry film as the photoresist material with a thickness of 100 μm (SUEX K100, K1 Solution, Gwangmyeong, Korea). The SU-8 film was laid between a metal substrate and the mask. For UV light exposure, a UV source with a wavelength of 362 nm and intensity of 10 mW/cm^2^ was used and we exposed the aluminum mask to UV light patterned with characters of “KFRI” for 20 min in a sealed case. The metal mask was fabricated with a size of 7 × 8 mm for each character and the line width was 1.5 mm. After UV treatment, we cooled the sample down to remove the thermal effect and measured its THz characteristics. Figure 1 shows the scheme for the UV light treatment process on an SU-8 dry film. 

### 2.2. THz Time-Domain Spectroscopy and Reflective Imaging Measurement

The terahertz time-domain spectroscopy (THz-TDS) system consists of two parts—generation and detection. A mode-locked Ti: sapphire femto-second pulsed laser produced a pulse width of 90 fs and center wavelength of 800 nm. The output laser beam was divided into two paths by a beam splitter for pumping and probing THz waves. To emit THz waves, a photoconductive antenna (PCA) was adopted. The PCA was switched using the conventional method to produce THz waves. When the ultra-fast laser excites a biased PCA circuit, THz pulses can be generated by the photocurrent. The THz photoconductive emitter PCA was fabricated on a GaAs substrate and the antenna pattern was a gold dipole antenna. The coherent detection method for THz waves is similar to the THz generation processing. The emitted THz pulses were acquired with a time-domain sweep at the PCA detector. Further, the gating DC signal from the THz detector can be monitored by photoconductive sampling using a lock-in amplifier. In this study, we used a commercial THz-TDS system (TPS-3000, Teraview, Cambridge, UK).

Reflective THz imaging was performed by using a two-dimensional scanning module on the THz-TDS. To avoid water vapor absorption, the whole reflection system (including THz-TDS) was filled with dry gas with less than 1% relative humidity. The angle between the incident and reflected signal of the focused THz beams was 30°. The focused beam diameter of the THz pulse was approximately 1 mm and the stage traveled in steps of 100 μm. We acquired the THz pulse for the time domain per pixel, and the acquisition time was 30 ms per pixel.

### 2.3. THz Signal Data Processing

Optical parameters, such as complex refractive index and absorption coefficient of a material, can be extracted by THz-TDS. The complex refractive index represented by $\tilde{n}=n_{1}+in_{2}$ is related to the phase delay of the THz pulse and absorbance in the materials compared to the reference signal (without the material). In other words, refractive index $n_{1}$ can be extracted from the phase delay, and $n_{2}$ is strongly related to the absorbance. The output THz signal *O*(*ω*) passing through a material is related to the input THz signal *I*(*ω*), regarded as the reference signal, as [21]

(1) $$O\left(\omega \right)=I\left(\omega \right)\exp\left[-\frac{d\alpha \left(\omega \right)}{2}\right]\exp\left[i\frac{2\pi }{\lambda }n_{1}\left(\omega \right)d\right],$$

where *α*(*ω*) is the frequency-dependent absorption coefficient, *d* is the thickness of the sample, and *λ* is the wavelength. The absorption coefficient *α*(*ω*) can be obtained from the difference between spectral amplitudes with and without the sample after Fourier transformation. It is related to the imaginary part of the complex refractive index *n*_2_ by

(2) $$\alpha \left(\omega \right)=-\frac{2}{d}ln\left[\frac{O\left(\omega \right)}{I\left(\omega \right)}\right]=\frac{4\pi n_{2}}{\lambda }.$$

In addition, *n*_1_ is measured from the difference of the THz signal phase between the with- and without-sample cases:

(3) $$n_{1}\left(\omega \right)=1+\frac{\Phi_{I}-\Phi_{O}}{2\pi d}\lambda ,$$

where Ф*_I_* and Ф*_O_* are the phases of the input (without sample) and output (with sample) pulses, respectively, throughout the sample.

## 3. Results and Discussion

**Figure 2:**
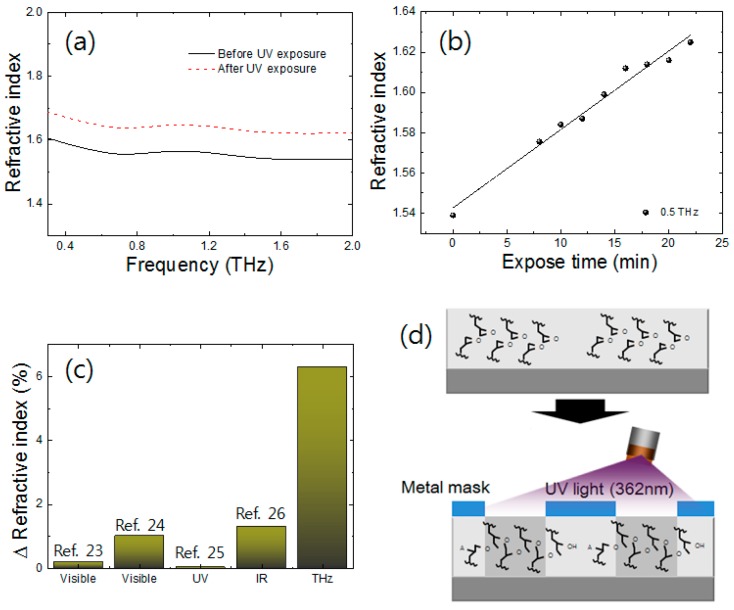
The refractive index on SU-8: (**a**) before and (**b**) after UV light (362 nm) treatment with an exposure time of 20 min; (**c**) The refractive index change ratio of SU-8 after UV exposure at visible, UV (351 nm), IR (1550 nm), and THz (0.5 THz) regions; (**d**) scheme of chemical change in SU-8 after UV light treatment.

At first glance, we measured the refractive index of SU-8 before and after UV exposure in the frequency range of 0.3–2 THz. We measured the refractive index of the SU-8 film without any treatment. To investigate the effect of UV light on SU-8, we exposed the same SU-8 film to UV light with a wavelength of 362 nm for 20 min. As shown in Figure 2a, there is a significant change in the refractive index of the SU-8 film after UV light exposure. Generally, when SU-8 is exposed to UV light, photon energy is absorbed into the SU-8 film and the photochemical reaction produces an acid [22]. Further, the acid acts as a catalyst during the post-baking exposure. This process promotes a cross-linking between epoxy groups and creates a three-dimensional network. Consequently, cross-linking by UV light causes the SU-8 film to contract and the chemical structure of SU-8 become denser compared to that before UV treatment. This phenomenon affects the change in the refractive index in the THz frequency region significantly. Figure 2c shows the scheme of chemical structural change in SU-8 after UV treatment. Figure 2b shows the exposure-time-dependent refractive index of the SU-8 film at 0.5 THz. The refractive index increases with UV exposure time. This result explains that as the UV light exposure time is increased, the cross-linking is also increased between epoxy groups in the SU-8 material. After UV treatment for 20 min, the refractive index increases up to a value of 1.636. In addition, the refractive index of SU-8 increases linearly. The refractive index change in SU-8 in various regions—such as visible, UV, and infrared (IR)—has been previously investigated [23,24,25,26]. Ong et al. investigated the refractive index of SU-8 in the visible region. In this investigation, an SU-8 film was treated with UV light having an intensity of 15 mW/cm^2^ with a time interval up to 40 min. Accordingly, the refractive index of the SU-8 film changed less than 0.2% compared to that of the non-treated SU-8 film at visible region. Salazar-Miranda et al., have reported a refractive index change of less than 1% in SU-8 in the visible region after UV treatment. In addition, the refractive index change of SU-8 has been reported with a very small variation in the UV (351 nm) and 1.3% in the IR region. In our results, in contrast, the refractive index of SU-8 changed by approximately 6% more than that before UV treatment in the THz frequency range. Consequently, the refractive index of SU-8 is strongly sensitive at THz frequencies, more than in the visible, UV, and IR regions.

**Figure 3:**
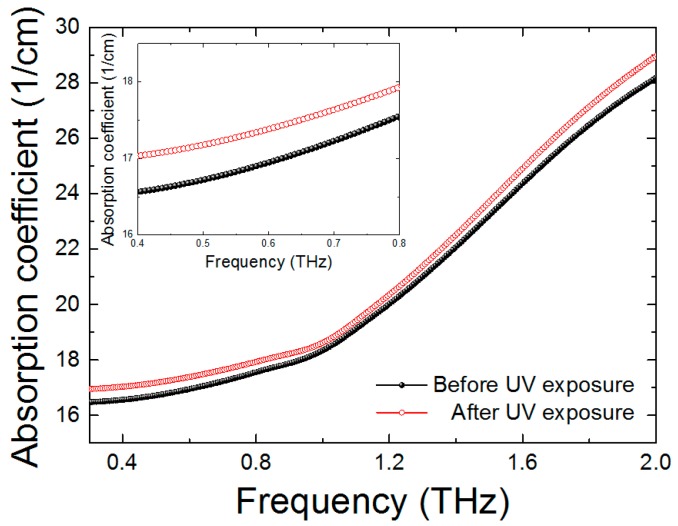
The absorption coefficient of the SU-8 film before and after UV exposure.

The absorption coefficient of the SU-8 film is measured before and after UV exposure as can be seen in Figure 3. The absorption coefficient is increased after UV treatment. From this result, the absorbance of SU-8 is enhanced by 2–3% at the measured THz range. It can be inferred that due to cross-linkages from UV treatment, THz absorbance is increased.

**Figure 4:**
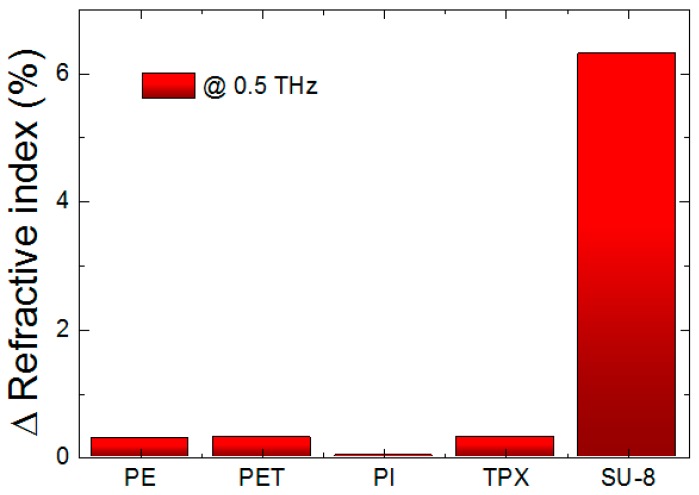
The change ratio of the refractive indices of polyethylene (PE), polyethylene terephthalate (PET), polyimide (PI), polymethylpentene (TPX), and SU-8 at 0.5 THz.

To compare with non-photoresist polymers for the enhancement of the refractive index change, we measured the refractive index of several representative non-photoresist polymers at 0.5 THz. The polymers for investigating THz characteristics were polyethylene (PE), polyethylene terephthalate (PET), polyimide (PI), polymethylpentene (TPX), and SU-8 films. The UV light exposure time was kept constant at 20 min for the samples. Figure 4 shows the ratio of change in the refractive index of each polymer at 0.5 THz. As can be seen in the figure, the refractive index of polyethylene, polyethylene terephthalate, polyimide, and polymethylpentene did not change significantly. These polymers can be effective under only 0.25% enhancement. In the case of SU-8, in contrast, its refractive index can be changed by more than 6%. The SU-8 material is affected by UV treatment approximately 24 times more compared with other non-photoresist polymers in the THz region. From this result, it can be inferred that a photoresist polymer is very sensitive in the THz region compared to non-photoresist polymers, and is suitable for THz security printing.

**Figure 5:**
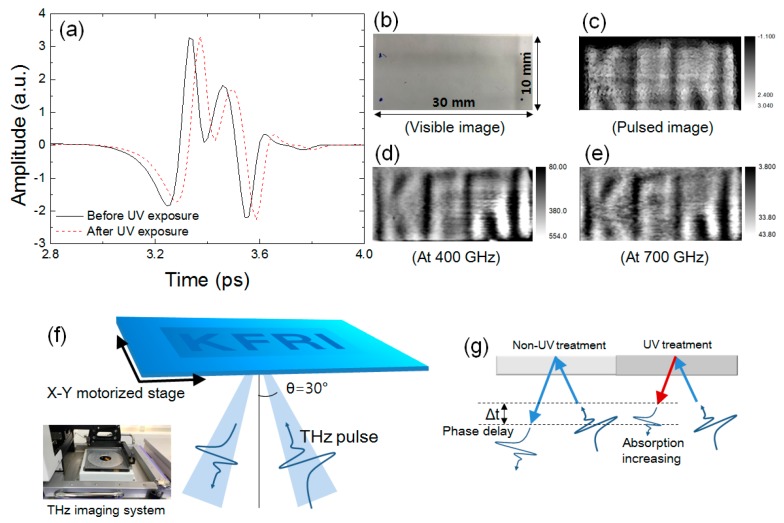
(**a**) The reflective THz pulse at UV non-treated and treated SU-8 area. The images of characters patterned on SU-8 film by (**b**) optical; (**c**) reflective THz pulses; (**d**) 400 GHz and (**e**) 700 GHz frequency; (**f**) an experimental scheme and real image of THz reflective imaging; (**g**) schematic of phase delay and absorption before and after UV treatment.

Finally, we investigated the THz image of a character-patterned SU-8 film by UV exposure using a reflective imaging system with THz spectroscopy. To obtain the THz image of the character that is patterned on the SU-8 film, we produced a metal mask patterned as “KFRI” and laid it on the SU-8 film. Then we exposed the SU-8 film to UV light for 20 min. The sample was moved by a two-dimensional X–Y stage and the THz images were obtained with a stage resolution of 100 μm and linear velocity of 30 ms per pixel, as shown in the real image of the THz imaging system shown in Figure 5f. The UV-printed SU-8 film imaged by the THz system is shown in Figure 5b. As can be seen in this figure, the “KFRI” characters are not shown as a visible image. However, the characters can be shown using THz pulses. Figure 5a represented the reflective THz pulsed images reflecting the UV-treated and non-UV-treated SU-8 film. The experimental scheme is shown in Figure 5f after UV light exposure; the phase of the THz pulse is delayed because the refractive index of SU-8 is increased as shown in Figure 5g. This phase delay causes a contrast change in the THz image. Hence, the characters “KFRI” can appear significantly, as shown in Figure 5c. If the sample is thin enough, that is, the time of the THz pulse passing through the sample is shorter than the pulse width of the THz pulse, the Fabry–Pérot effect affects the THz pulse due to multiple reflections. In this case, however, the peak of the THz pulse at time-domain can be separated with a reference THz signal regardless of the Fabry–Pérot effect. Hence, the THz pulsed image can be obtained successfully. Moreover, if the THz pulse with a short pulse width less than the thickness of the sample can be generated, the THz pulse would be obtained without multiple Fabry–Pérot reflections.

In addition, the cross-linking between the epoxy groups could enhance the structural density of SU-8. This processing causes increased absorbance in the THz region (Figure 5f). This result shows that the changed absorbance can also cause the image contrast. From this, we can read the image of the character-patterned SU-8 at a single frequency of 400 and 700 GHz, as shown in Figure 5d,e. Accordingly, the characters can be observed prominently at a single frequency. In the THz pulse and single-frequency images, gradation of values in the images can be seen. This is because the SU-8 film is slightly rumpled after UV treatment. When the SU-8 film is exposed to UV light, the UV intensity decreases with the depth of the SU-8 surface. Consequently, the density changes at each depth of the film. Hence, the path of the THz pulse can deviate at the boundary between the UV-exposed and normal area. This causes the contrast gradation in THz images. 

## 4. Conclusions

We studied the THz characteristics of SU-8 as a photoresist polymer and its applications to security printing and anti-forgery. We measured the refractive index of an SU-8 film by UV light exposure time dependency in the THz range. The refractive index of SU-8 changed significantly as the UV exposure time increased. It is due to the change in the chemical structure after UV exposure by cross-linking between epoxy groups. Consequently, the value of the refractive index increased from 1.539 to 1.636 at 0.5 THz. However, there was no significant change in other non-photoresist polymers such as polyethylene, polyethylene terephthalate, polyimide, and polymethylpentene. To demonstrate the security application, we also obtained the THz image of characters patterned on the SU-8 film by using reflective THz pulses. The THz pulse and THz single frequency can be used to distinguish images between UV treatment and non-treatment successfully, but the characters cannot be seen in the visible image. It can be concluded that SU-8 is a good candidate for security applications in the THz range. Consequently, these results can be applied to currency, passport, food packaging, and other areas by using a simple method consisting of a photoresist polymer, UV light, and THz system. For future work, to apply this technique to security and anti-forgery, we plan to improve the scanning time and image resolution.
